# Analysis of suicides in one of the administrative regions of Bulgaria for 10-year period (2009-2018)

**DOI:** 10.1093/eurpub/ckac131.484

**Published:** 2022-10-25

**Authors:** I Veleva, K Stoychev, M Stoimenova, E Mineva

**Affiliations:** 1 Psychiatry and Medical Psychlogy, Medical University of Pleven, Pleven, Bulgaria; 2 Department of Public Health Sciences Medical University of Pleven, Pleven, Bulgaria

## Abstract

**Background:**

Suicide is an important medical and social problem responsible for nearly one million deaths per year globally. However, distal and proximal risk factors for suicide, expect being mentally diagnosed, are not enough studied. The aim of the study was to analyze the dynamics, structure, socio-demographic and clinical characteristics of all suicides committed by persons with mental disorder for 10-year period (2009-2018) in Pleven district.

**Methods:**

Retrospective analysis of medical records of all mentally ill persons who committed suicide was done. Data were extracted from the databases of all in- and outpatient mental health centers in the region. Data processing was performed by IBM SPSS Statistics v.25. Statistical associations between a number of socio-demographic and clinical characteristics and the age of suicide victims was studied by dispersion analysis and Mann-Whitney test. Statistical significance was set at p ≤ 0.05.

**Results:**

Among all 281 registered suicide cases during the studied period, 77 (28%) were with mental disorders. The most common were mood disorders (44%), followed by schizophrenia, anxiety disorders, substance abuse disorders and organic mental conditions. The mean age of all suicides was 55.62 years; significantly lower in males than in females (p = 0.042); lower in divorced or never married/single living persons compared to married or who had lived with a partner (F (2.74) =17.682, p < 0.001]; the lowest in patients with schizophrenia (44.62 years), and the highest in organic disorders (66.83 years). Higher educational degree was associated with lower age of suicide (U = 3.713, p < 0.01) and the earlier age of onset of the psychiatric disorder (r = 0.754, p < 0.001). Most of the suicide cases had occurred in March and September. Tuesdays and Fridays were most suicidal.

**Conclusions:**

Severe mental disorders are major risk factors for suicide with the additional contribution of certain socio-demographic and disease related characteristics.

**Key messages:**

• Suicide monitoring should be constant in all patients with chronic and severe mental disorders.

• Suicide registration in Bulgaria needs to be improved in terms of collecting sufficient and reliable information about the mental health of suicide victims.

